# Effectiveness of antenatal care services in reducing neonatal mortality in Kenya: analysis of national survey data

**DOI:** 10.1080/16549716.2017.1328796

**Published:** 2017-06-16

**Authors:** Malachi Arunda, Anders Emmelin, Benedict Oppong Asamoah

**Affiliations:** ^a^ International Master Programme in Public Health, Faculty of Medicine, Lund University, CRC, Malmö, Sweden; ^b^ Social Medicine and Global Health, Department of Clinical Sciences, Lund University, Malmö, Sweden

**Keywords:** Antenatal care, neonatal mortality, tetanus toxoid, population attributable fraction

## Abstract

**Background**: Although global neonatal mortality declined by about 40 percent from 1990 to 2013, it still accounted for about 2.6 million deaths globally and constituted 42 percent of global under-five deaths. Most of these deaths occur in developing countries. Antenatal care (ANC) is a globally recommended strategy used to prevent neonatal deaths. In Kenya, over 90 percent of pregnant women attend at least one ANC visit during pregnancy. However, Kenya is currently among the 10 countries that contribute the most neonatal deaths globally.

**Objective**: The aim of this study is to examine the effectiveness of ANC services in reducing neonatal mortality in Kenya.

**Methods**: We used binary logistic regression to analyse cross-sectional data from the 2014 Kenya Demographic and Health Survey to investigate the effectiveness of ANC services in reducing neonatal mortality in Kenya. We determined the population attributable neonatal mortality fraction for the lack of selected antenatal interventions.

**Results**: The highest odds of neonatal mortality were among neonates whose mothers did not attend any ANC visit (adjusted odds ratio [aOR] 4.0, 95% confidence interval [CI] 1.7–9.1) and whose mothers lacked skilled ANC attendance during pregnancy (aOR 3.0, 95% CI 1.4–6.1). Lack of tetanus injection relative to one tetanus injection was significantly associated with neonatal mortality (aOR 2.5, 95% CI 1.0–6.0). About 38 percent of all neonatal deaths in Kenya were attributable to lack of check-ups for pregnancy complications.

**Conclusions**: Lack of check-ups for pregnancy complications, unskilled ANC provision and lack of tetanus injection were associated with neonatal mortality in Kenya. Integrating community ANC outreach programmes in the national policy strategy and training geared towards early detection of complications can have positive implications for neonatal survival.

## Background

Antenatal care (ANC) is one of the key strategies recommended to reduce the risk of neonatal mortality in any community irrespective of socio-demographic background [1–4]. Although global neonatal mortality (deaths before 28 days of age) declined by 40 percent from 1990 to 2013 [5,6], it still accounted for about 2.6 million deaths globally and constituted 42 percent of global under-five mortality (deaths before 5 years of age) [7]. Over 99 percent of neonatal mortalities occur in low- and middle-income countries [8]. While the absolute numbers of under-five deaths have declined, the proportion of deaths occurring in the neonatal period has risen and this is because of the slower declining rates of neonatal mortalities [9]. From 1990 to 2013, the neonatal proportion of under-five mortality increased from 37 to 44 percent [5]. Reducing global under-five mortality is thus largely dependent on a reduction in neonatal deaths [3]. In sub-Saharan Africa (SSA), high numbers of neonatal deaths are sustained by the high fertility rate of about 5.1 births per woman [10].

In many SSA countries, the leading risk factors for neonatal deaths include preterm births, birth complications, infections such as tetanus, sepsis and pneumonia [1,11,12]. All these risk factors can be minimized or prevented through ANC interventions. The World Health Organization (WHO) recommendation for effective ANC services, specific to low-income countries, is four or more ANC visits [13]. The recommendation requires each of the first two ANC visits to take place in the first two trimesters and the last two visits should take place in the last trimester [13]. Each visit is required to focus on a given ANC service package as outlined in the WHO guidelines [13]. Overall, the main services include screening for complications, health education for healthy lifestyle, 2 tetanus toxoid (TT) injections and 90 iron/folic acid (IFA) tablets [13]. Several countries have adopted these recommendations and the coverage of at least one ANC visit has been high.

However, while the decline in neonatal mortality rate (NMR) has progressed moderately fast in some low-income countries, in others, the decline has been slow [9,14]. For instance, the NMR in Rwanda and Cambodia declined from 42 to 19 and 35 to 15 (per 1000 live births) between 2000 and 2015 respectively, while in Kenya the NMR declined from 29 to 22 during the same period [14].

Kenya is among the 10 countries that contribute the most neonatal deaths globally [15]. In addition to the WHO recommendations, the Kenyan government introduced free maternal health services that included antenatal and delivery care in first-level government health facilities from June 2013 [16].

Like in many other SSA countries, estimating the impact of ANC in improving neonatal or birth outcomes in Kenya has been difficult [17]. Most studies have focused on the effectiveness of coverage and implementation of ANC services [18–21] but there exists no thorough standardized system to evaluate the interventions in terms of neonatal survival. A number of community and health facility-based studies in SSA have investigated the effectiveness of various components of ANC interventions in reducing neonatal mortality [22,23]. However, these studies have been limited to the fraction of births that are registered in the health facilities and neonatal deaths that are inadequately reported and are therefore less representative of the entire population. A few similar population-based or nationally representative studies have also been conducted in some SSA countries [20,21,24,25]. In 2011, McCurdy et al. [26] conducted an aggregate-level study in the region, which involved 17 least developed SSA countries, excluding Kenya [26]. The study generally found that ANC from a skilled provider was associated with decreased risk of neonatal mortality. The study also identified that the most effective ANC interventions included TT injection, and weight and blood pressure (BP) measurements [26]. However, with the very wide disparities in NMRs among the countries observed in the study and the wider differences in the years of surveys for the various countries included (between 2003 and 2009), country-specific analysis would provide more comprehensive and reliable findings at the country level.

The aim of this study is to examine the effectiveness of ANC services in reducing neonatal mortality in Kenya. The findings will highlight the impact of ANC interventions, with implications to improve health workers’ operations, cost-effectiveness and to accelerate the reduction of neonatal deaths.

## Methods

### Study settings

The Kenyan population was estimated to be 47.8 million in 2015 [27] and about 75 percent live in the rural areas [28]. With the fertility rate at 4.1 and sex ratio of 1:1, an estimated 22,000 births occur every day [27]. Like many SSA countries, agriculture is the main economic activity [29]. In June 2013, the Kenyan government introduced free maternal and child health services in all first-level public health facilities and this resulted in an increase in the number of pregnant women using ANC services and about an 18 percent increase (from 44 percent in 2009 to 62 percent in 2014) in skilled attendance during delivery at the health facilities [30]. The main challenges affecting maternal and child health care in Kenya are severe in rural areas and include inadequately skilled health providers, distance to the health facilities (in rural areas), burden of HIV/AIDS, and insufficient facilities, equipment and supplies[16,31].

### Study design and data source

We obtained cross-sectional survey data from the 2014 Kenya Demographic and Health Survey (DHS). The DHS randomly samples data across the entire country and in all the counties. The 2014 Kenya DHS used a sampling frame generated from the National Sample Survey and Evaluation Programme (NASSEP V) and therefore the data-sets are nationally representative [32]. The survey used household and individual (mothers) questionnaires to collect information on mortality, ANC, family planning, reproduction and socio-demographic characteristics [32]. In this study, only the most recent singleton children, born 1–59 months (~5 years) prior to the 2014 survey, were included in the study. This improved the accuracy of verbal interview since the respondents (mothers) could easily recall the most recent birth occurrences or readily provide the ANC card for reference, thus strengthening the internal and external validity of this study. Further, singleton selection eliminated any confounding effect of multiple births that are prone to mortality due to biological factors other than inadequate ANC. A total of 14,190 cases were included in the study. The participants remained anonymous. The mothers were asked a series of structured, direct, probing and follow-up questions on reproduction and various ANC services they had received or not received. The responses were filtered and coded into two or more categories [33]. The DHS methodology toolkit and field manuals are available for further details on data sampling and collection methods [34,35].

### Study variables

#### Outcome variables

*Neonatal mortality*: This was defined as the death of a baby before reaching 28 days (one month) of age. This variable was dichotomized into yes (for neonates who died within a month) and no (were alive for the first month of life).

#### Primary predictor variables

*ANC visits*: This meant the number of times an expectant mother visited a skilled provider for check-ups and pregnancy-related advice during pregnancy until the delivery of the baby. It was classified into no visit, 1–3 visits and 4 or more visits.

*TT injection*: TT vaccination protects the mother and the baby against tetanus, a deadly infection caused by *Clostridium tetani* bacteria which enter the body through skin cuts and wounds such as those during delivery or cutting of the umbilical cord [36,37]. In this study, we examined the effectiveness of no tetanus injection and two or more tetanus injections relative to one in preventing neonatal deaths.

*IFA*: Intake of IFA supplements is recommended to lower the risk of preterm birth, low birth weight, anemia and subsequently neonatal mortality [38]. In the present study, we compared the effect of 90 or more IFA tablets, as recommended by the WHO, and less than 90 in improving neonatal survival.

*ANC assistance*: Skilled assistance included ANC attendance conducted by doctors, nurses, clinical or medical officers while unskilled assistance included those done by traditional birth attendants (TBA), nursing aids, community health workers (CHW), relatives, friends and others. This classification was similar to that of birth attendance.

Other ANC services examined in this study include urine analysis [39], checking of BP [40], screening of weight, blood tests and check-ups for pregnancy complication.

#### Socio-demographic, maternal and birth-related variables

These comprised independent variables that are theorized to be non-causal risk factors to neonatal mortality. Some variables are also associated with lack of or inadequate ANC and are included in this study as confounding variables. They include place of residence (rural), birth attendance, cesarean birth [41], parity [42,43], advanced maternal age [42], low birth weight [43,44], poverty (low wealth) [45], low education level [46] and single motherhood [47]. The wealth status classification of the DHS is based on a composite measure of a household’s cumulative living standard measured in terms of household assets such as cars, bicycles, type of water and sanitation facility used and housing construction materials [48]. The wealth index is computed based on the asset index of socioeconomic status [48]. It also takes into account the rural and urban settings. A statistical procedure known as principal components analysis is then used to categorize individual households on a continuous scale of relative wealth [48]. In this study, single women included those who were never married, widowed, divorced or separated and not living together while married women were those who were married or living together at the time of neonatal death. Similarly, education level of less than 9 years was primary level and 9 years or more was secondary or higher level while no formal education was referred to as no education. Descriptions and classifications of the study variables are detailed in Box 1.

### Box 1. Summary of variables

**Table:** 

Variable category	Variable	Categorization
Outcome variable	Neonatal mortality	Yes (Died within 1 month)
		No (Was alive for the first month of life)
Predictor variables	ANC visits	No visit
		1‒3 ANC visits
		4 or more (≥ 4) ANC visits
	ANC attendance	Unskilled attendant
		Skilled attendant
	Timing of 1^st^ ANC visit	1^st^ trimester
		2^nd^ trimester
		3^rd^ trimester
	Tetanus toxoid (TT) injection(s)	No injection
		1 TT injection
		2 or more (≥ 2) TT injections
	Iron/folic acid (IFA)	0 to < 90 tablets
		90 or more (≥ 90) tablets
	Birth attendance	Unskilled attendant
		Skilled attendant
	Weight screened, BP	Yes and
	checked, analysis of	no
	blood/urine done	
Socio-demographic/	Maternal age	15–24, 25–34 and 35–49
maternal/birth-	Low birth weight (LBW)	Yes (< 2500 g)
related variables		No (≥ 2500 g)
	Cesarean birth	Yes or no
	Parity	Nulliparous, para 1–3 and
		para 4+
	Marital status	Single and married
	Maternal education level	No or primary education
		Secondary or higher
	Wealth index	Poor, middle and rich
	Place of residence	Rural and urban
*Parity* refers to the number of times a woman has given birth to a fetus of 24 or more weeks gestation age, irrespective of whether the child is dead or alive [42]. *LBW* is birth weight less than 2500 grams [49].		

### Statistical methods

Pearson’s chi-square test of independence was employed to examine the distribution of ANC interventions and neonatal survival status. Binary logistic regression was used to determine both the crude and adjusted odds ratios (cOR and aOR, respectively) for the associations between lack of or inadequate ANC services and neonatal mortality. Statistical significance was established at 95 percent confidence intervals (95% CI). Data sampling weights were applied and the complexity of the DHS sampling design was taken into account in order to improve the representativeness of the data. To eliminate the effect of potential confounding, stepwise regression modeling was used. All the socio-demographic variables, maternal and birth-related variables were considered as potential confounders in this study. Possible associations between each of the predictor variables, each of the other independent variables and the outcome variable (neonatal mortality) were assessed by observing the *p*-values. Only predictor variables that indicated possible associations (*p*-value < 0.05) with the dependent variable (neonatal mortality) were subjected to further analysis. All the other predictor variables were excluded from further analysis. Crude odds ratios were generated in model 1. In model 2, adjusted odds ratios for the associations between lack of or inadequate ANC interventions and neonatal mortality were determined after controlling for maternal background variables (socio-demographic). In model 3, in addition to the confounding variables in model 2, the model adjusted for birth-related variables (skilled birth attendance and cesarean birth). Model 4 adjusted for all the confounding variables in models 2 and 3 plus LBW. All the socio-demographic, maternal and birth-related variables were also adjusted for each other in each of the models where they were included in the analysis. We used the statistical software package IBM SPSS version 22.0 (IBM, Armonk, New York, USA) for analysis and Microsoft Excel to graphically summarize the effectiveness of ANC interventions in reducing neonatal mortality.

#### Estimation of population attributable risk fraction of selected ANC interventions

The population attributable neonatal mortality risk fraction (PAR) for lack of or inadequate ANC services was computed as the fraction of neonatal deaths that could have been prevented if the given ANC service had been provided. The PAR was determined using the formula,


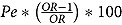
, where OR is the odds ratio, adjusted for all the confounding variables in the study in the logistic regression analysis, and *Pe* is the proportion of deaths that lacked the given ANC service, thus exposed.

## Results

Table 1 shows the distribution of maternal background characteristics by ANC services and birth attendance. Over 95 percent of all the pregnant women in Kenya aged 15–49 had at least one ANC visit during the 1‒59 months (~5 years) prior to the 2014 survey. About 89 percent had at least one TT injection. About 20 percent of the women had their first ANC visit in the first trimester and over 63 percent of all the mothers in the survey had skilled birth attendance for their most recently born child.

**Table 1.** Distribution of maternal background characteristics, by antenatal care (ANC) services and birth attendance.

	At least 1 ANC visit, N = 14,144			At least 1 TT, N = 6703			Given/bought 90 or more IFA, N = 13,912			1^st^ ANC visit in 1^st^ trimester, N = 13,595			Skilled birth assistance, N = 14,189		
Variables	Yes (%)	No (%)	χ^2^ p	Yes (%)	No (%)	χ^2^ p	Yes (%)	No (%)	χ^2^ p	Yes (%)	No (%)	χ^2^ p	Yes (%)	No (%)	χ^2^ p
Education level															
No education	84.5	15.5	< 0.01	76.6	23.4	< 0.01	1.8	98.2	< 0.01	15.4	84.6	< 0.01	28.8	71.2	< 0.01
Primary	96.3	3.7	< 0.01	89.2	10.8	< 0.01	3.3	96.7	< 0.01	16.8	83.2	< 0.01	59.7	40.3	< 0.01
Secondary/higher	98.8	1.2		94.6	5.4		4.4	95.6		27.4	72.6		86.8	13.2	
Marital status															
Single	94.3	5.7	< 0.01	90.0	10.0	> 0.05	2.9	97.1	< 0.05	19.6	80.4	> 0.05	69.0	31.0	< 0.01
Married	96.4	3.6		89.8	10.2		3.7	96.3		20.6	79.4		65.7	34.3	
Place of residence															
Rural	94.7	5.3	< 0.01	87.9	12.1	< 0.01	2.9	97.1	< 0.01	17.1	82.9	< 0.01	55.1	44.9	< 0.01
Urban	98.1	1.9		93.0	7.0		4.6	95.4		26.0	74.0		84.3	15.7	
Wealth index															
Poor	92.7	7.3	< 0.01	85.9	14.1	< 0.01	2.9	97.1	< 0.01	15.7	84.3	< 0.01	43.7	56.3	< 0.01
Middle	97.5	2.5	< 0.01	89.2	10.8	< 0.01	2.8	97.2	< 0.01	17.3	82.7	< 0.01	65.6	34.4	< 0.01
Rich	98.5	1.5		93.9	6.1		4.5	95.5		26.3	73.7		88.1	11.9	
Parity															
Nulliparous	97.4	2.6		94.6	5.4		3.4	96.6		26.4	73.6		84.6	15.4	
Para 1–3	97.0	3.0	< 0.05	92.9	7.1	< 0.05	4.0	96.0	> 0.05	19.9	80.1	< 0.01	67.9	32.1	< 0.01
Para 4+	93.1	6.9	< 0.01	81.6	18.4	< 0.01	2.5	97.5	> 0.05	15.1	84.9	< 0.01	42.1	57.9	< 0.01
Maternal age															
15–24	96.2	3.8	0.6	92.8	7.2	< 0.01	3.3	96.7	> 0.05	19.8	80.2	0.001	62.2	36.8	< 0.01
25–34	96.5	3.5		90.7	9.3		3.7	96.3		22.1	77.9		60.5	39.5	
35–49	94.4	5.6	< 0.01	83.6	16.4	< 0.01	3.4	96.6	> 0.05	18.0	82.0	< 0.01	51.5	48.5	< 0.01
Average (%)	**95.4**	**4.6**		**89.1**	**10.9**		**3.4**	**96.6**		**20.2**	**79.8**		**63.5**	**36.5**	

Notes: χ^2^ p – *p*-value from chi-square test. TT – tetanus toxoid injection. IFA – iron folic/acid. < 90 IFA includes those who had zero.

Table 2 shows the distribution (including *p*-values) of all the study variables by neonatal survival status and NMR. The NMR was four times higher among those who did not attend any ANC visit relative to those who had four or more visits. About 67% of all the expectant mothers in the survey had their first ANC visit in the second trimester and more than half of all the women had four or more ANC visits during pregnancy. The NMR was about 3.5 times higher among neonates whose mothers had unskilled ANC attendance relative to those whose mothers had skilled ANC attendance. Similarly, mothers who did not receive any pregnancy complication check-up had a 2.5 times higher NMR compared to those who received a check-up. The NMR was twice higher among neonates whose mothers had no TT injection as compared to those who had one TT injection. Although the NMRs were comparatively higher among neonates whose mothers had no or fewer than 90 IFA tablets, no BP check and no blood tests or urine analysis, there was no statistically significant associations (*p* ˃ 0.05) detected between the lack of these ANC interventions and neonatal mortality.

**Table 2.** Distribution of all the study variables by neonatal survival status in Kenya, neonatal mortality rates (NMR) and *p*-values.

	Neonatal survival status (percent)			
Variables	Died (N = 191)	Alive (N = 13,997)	NMR (per 1000 live births)	*p*-value
**ANC visits**				
No visit	24 (12.6)	539 (3.9)	42.6	< 0.01
1–3 visits	80 (41.9)	5340 (38.2)	14.8	< 0.05
4 or more visits	87 (45.5)	8074 (57.9)	10.7	
**First ANC visit**				
1^st^ trimester	31 (18.6)	2766 (20.6)	11.1	
2^nd^ trimester	118 (70.7)	8959 (66.7)	13.0	> 0.05
3^rd^ trimester	18 (10.8)	1704 (12.7)	10.5	> 0.05
**ANC attendant**				
Unskilled	26 (13.6)	555 (4.0)	44.8	< 0.001
Skilled	165 (86.4)	13,442 (96.0)	12.1	
**TT**				
No TT injection	15 (18.1)	665 (10.0)	22.0	< 0.01
1 TT injection	29 (31.2)	2739 (39.3)	10.5	
≥ 2 TT injections	36 (48.4)	3405 (48.6)	13.1	> 0.05
**Bought/given IFA**				
0 to < 90 IFA	182 (96.3)	13,237 (96.5)	13.6	> 0.05
90 or more	7 (3.7)	485 (3.5)	14.2	
**BP checked**				
No	6 (8.0)	390 (6.1)	16.2	> 0.05
Yes	69 (92.0)	6047 (93.9)	11.3	
**Urine analysis**				
No	11 (14.7)	723 (11.2)	15.0	> 0.05
Yes	64 (85.3)	5711 (88.8)	11.0	
**Blood tests**				
No	4 (5.3)	257 (4.0)	16.3	> 0.05
Yes	71 (94.7)	6179 (96.0)	11.4	
**Complications check**				
No	49 (65.3)	2668 (41.5)	18.0	< 0.001
Yes	26 (34.7)	3765 (58.5)	6.9	
**Birth attendant**				
Unskilled	70 (36.5)	4704 (33.6)	14.7	> 0.05
Skilled	122 (63.5)	9293 (66.4)	13.0	
**Cesarean birth**				
Yes	28 (14.7)	1300 (9.3)	21.1	< 0.05
No	163 (85.3)	12,691 (90.7)	12.7	
**Place of delivery**				
Health facility	117 (64.6)	8224 (63.9)	14.0	
Home/other places	64 (35.4)	4649 (36.1)	13.6	> 0.05
**Sex of the child**				
Male	94%	7152	13.0	> 0.05
Female	97%	6845	14.0	
**Parity**				
Para 1–3	83 (43.2)	7305 (52.2)	11.5	> 0.05
Para 4+	63 (32.8)	3129 (22.4)	19.5	> 0.05
Nulliparous	46 (24.0)	3563 (25.5)	11.7	
**Low birth weight**				
Yes (< 2500 g)	7 (8.3)	293 (4.4)	23.3	> 0.05
No (≥ 2500 g)	77 (91.7)	6370 (95.6)	11.9	
**Maternal age^1^**				
15–24	51 (26.7)	4192 (29.9)	12.0	> 0.05
25–34	80 (41.9)	6977 (49.8)	11.3	
35–49	60 (31.4)	2828 (20.2)	20.8	< 0.001
**Education level**				
No education	25 (13.1)	1366 (9.8)	18.0	< 0.01
Primary	119 (62.3)	7607 (54.3)	15.4	< 0.01
Secondary or higher	47 (24.6)	5025 (34.9)	9.3	
**Marital status**				
Single	34 (17.7)	2594 (18.5)	12.9	> 0.05
Married	158 (82.3)	11,404 (81.5)	13.7	
**Place of residence**				
Rural	114 (59.7)	8613 (61.5)	13.1	> 0.05
Urban	77 (40.3)	5384 (38.5)	14.1	
**Wealth index**				
Poor	89 (46.6)	5554 (39.7)	15.8	< 0.05
Middle	38 (19.9)	2557 (18.3)	14.6	> 0.05
Rich	64 (33.5)	5886 (42.1)	10.8	
**Sex of the child**				
Male	94 (49.2)	7152 (51.1)	13.0	> 0.05
Female	97 (50.8)	6845 (48.9)	13.9	
Sample neonatal mortality rate (NMR) = 1 3.5. NMR was calculated as (.				

Table 3 presents the binary logistic regression analyses (crude and adjusted odds ratios) for the associations between lack of or inadequate ANC services and neonatal mortality. Overall, after controlling for the effect of maternal background characteristics and birth-related factors including LBW (Model 4), the odds of neonatal death were four times higher among neonates whose mothers had no ANC visit relative to those whose mothers had four or more (aOR 4.0, 95% CI 1.7‒9.1). The neonates whose mothers had 1–3 ANC visits had about twice higher odds of deaths compared to those whose mothers had four or more ANC visits. Similarly, the odds of neonatal mortality were significantly higher among neonates whose mothers had no TT injection as compared to those whose mothers had only one TT injection (aOR 2.5, 95% CI 1.0‒6.0). There was no statistically significant difference in the odds of neonatal mortality between mothers who received two or more TT injections and those who received one TT injection (aOR 0.9, 95% CI 0.5‒1.4). Neonates whose mothers had unskilled ANC attendance had three times higher odds of mortality relative to those whose mothers had skilled ANC attendance (aOR 3.0, 95% CI 1.4‒6.1). Lack of check-up for complications during pregnancy was associated with neonatal mortality (aOR 2.4, 95% CI 1.5‒4.0). There was no statistically significant difference between neonatal survival rates when comparing skilled or unskilled birth attendance.

**Table 3.** Binary logistic regression analysis (models 1–4) showing the associations between antenatal care (ANC) interventions and neonatal mortality in Kenya, crude odds ratios (cOR) and adjusted odds ratios (aOR) with 95 percent confidence intervals (CI).

		Model 1		Model 2		Model 3		Model 4	
Variables /variable classifications	Categories	cOR	(95% CI)	aOR	(95% CI)	aOR	(95% CI)	aOR	(95% CI)
**ANC visits**	0 visits	4.2	(2.7–6.7)	3.9	(2.3–6.3)	4.4	(2.5–7.6)	4.0	(1.7–9.1)
	1–3 visit(s)	1.4	(1.0–1.9)	1.3	(1.0–1.8)	1.4	(1.0–1.9)	1.8	(1.1–3.0)
	4 or more	1.0	Ref.	1.0	Ref.	1.0	Ref.	1.0	Ref.
**ANC assistance**	Unskilled	3.8	(2.5–5.8)	3.4	(2.1–5.3)	3.7	(2.4–5.9)	3.0	(1.4–6.1)
	Skilled	1.0	Ref.	1.0	Ref.	1.0	Ref.	1.0	Ref.
**TT injection**	0 TT	2.4	(1.1–5.6)	2.4	(1.0–5.7)	2.3	(1.0–5.5)	2.5	(1.0–6.0)
	1 TT	1.0	Ref.	1.0	Ref.	1.0	Ref.	1.0	Ref.
	2 or more	0.9	(0.5–1.4)	0.9	(0.6–1.5)	0.9	(0.6–1.5)	0.9	(0.5–1.4)
**Complications check**	Yes	1.0	Ref.	1.0	Ref.	1.0	Ref.	1.0	Ref.
	No	2.7	(1.7–4.3)	2.4	(1.5–3.9)	2.4	(1.5–4.1)	2.4	(1.5–4.0)
**Maternal age**	15–24	1.1	(0.8–1.5)	1.0	(0.7–1.5)	1.2	(0.6–2.4)	0.9	(0.5–1.8)
	25–34	1.0	Ref.	1.0	Ref.	1.0	Ref.	1.0	Ref.
	35–49	1.8	(1.3–2.6)	1.7	(0.9–3.3)	1.5	(1.1–2.3)	2.1	(1.2–3.7)
**Education level**	No education	1.4	(1.1–1.8)	1.9	(1.3–2.9)	2.1	(0.9–4.5)	2.4	(0.8–6.6)
	Primary	1.6	(1.2–2.4)	1.2	(1.0–2.4)	1.3	(1.0–2.4)	1.2	(0.7–2.1)
	Secondary	1.0	Ref.	1.0	Ref.	1.0	Ref.	1.0	Ref.
**Parity**	Nulliparous	1.0	Ref.	1.0	Ref.	1.0	Ref.	1.0	Ref.
	Para 1–3	0.9	(0.6–1.3)	1.2	(0.7–2.1)	1.0	(0.5–2.0)	0.8	(0.3–2.5)
	Para 4+	1.6	(1.1–2.3)	1.4	(0.9–2.3)	1.2	(0.7–2.0)	1.5	(0.4–6.2)
**Place of residence**	Rural	1.1	(0.8–1.4)	1.2	(0.9–2.0)	1.5	(1.1–2.2)	1.4	(0.9–2.5)
	Urban	1.0	Ref.	1.0	Ref.	1.0	Ref.	1.0	Ref.
**Wealth index**	Poor	1.2	(0.9–1.7)	1.2	(0.8–2.1)	1.3	(1.0–1.9)	1.8	(1.0–3.6)
	Middle	1.4	(0.9–2.0)	1.3	(0.8–2.3)	1.3	(0.9–2.1)	1.6	(1.0–3.5)
	Rich		1.0	Ref.	1.0	Ref.	1.0	Ref.	1.0
**Cesarean birth**	Yes	1.7	(1.1–2.5)			1.6	(1.1-3.4)	1.4	(0.7–3.0)
	No	1.0	Ref.			1.0	Ref.	1.0	Ref.
**Birth assistance**	Unskilled	0.9	(0.7–1.2)			1.2	(0.7–2.1)	0.7	(0.6–1.5)
	Skilled	1.0	Ref.			1.0	Ref.	1.0	Ref.
**Low birth weight**	Yes (< 2500 g)	1.9	(0.8–4.2)					2.0	(0.9–4.5)
	No (≥ 2500 g)	1.0	Ref.					1.0	Ref.

Notes: Ref. – reference. **Model 1**: shows crude odds ratios. **Model 2**: Adjusted for maternal and socio-demographic variables. **Model 3**: Adjusted for maternal/socio-demographic variables plus birth-related variables (cesarean and skilled birth attendance). **Model 4**: Adjusted for maternal/socio-demographic variables, birth-related variables plus low birth weight. Variables in each model were mutually adjusted for each other.

**Figure 1.**
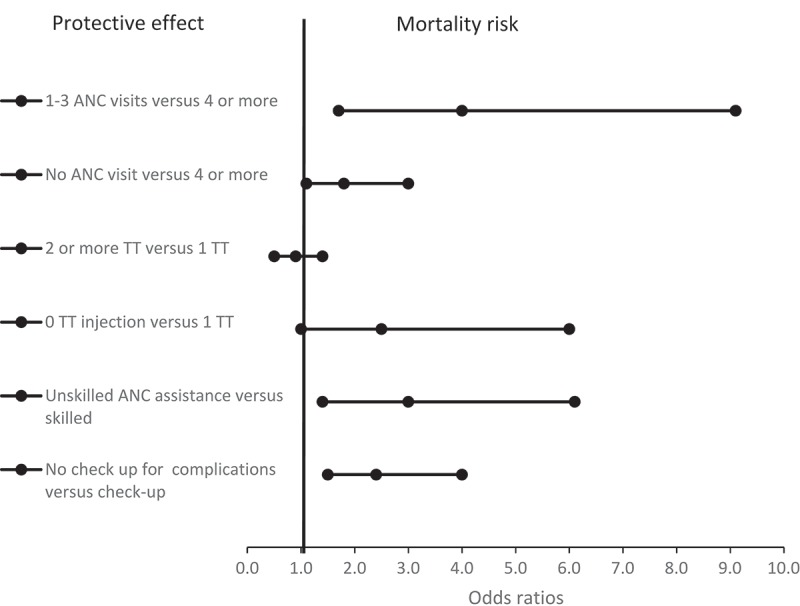
Graphical representation of model 4’s results showing adjusted odds ratios for the associations between ANC interventions and neonatal mortality.

**Figure 2.**
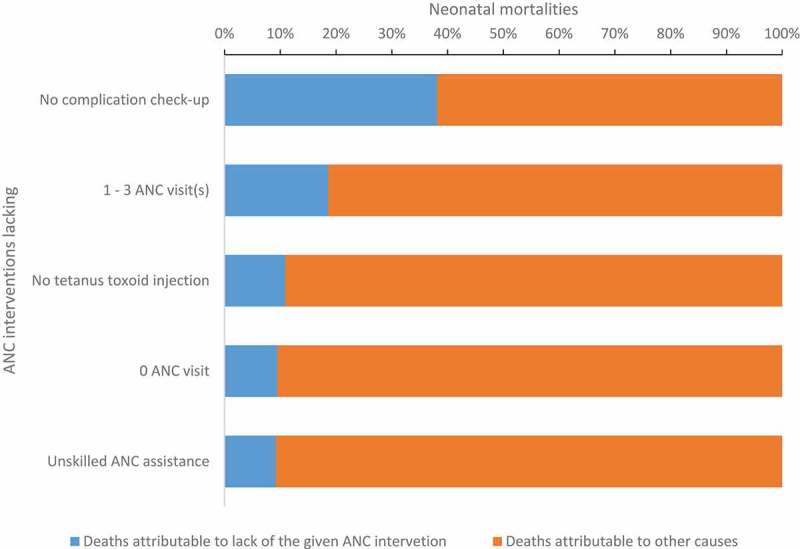
Population attributable neonatal mortality risk fraction for lack of or inadequate ANC interventions.

Figure 1 summarizes model 4’s results. It indicates that inadequate or lack of ANC visits, unskilled ANC attendance, no TT injection and lack of check-up for complications were associated with neonatal mortality in Kenya. Figure 2 is a graph showing that about 38 percent of neonatal deaths that occurred within the 5 years prior to the 2014 DHS survey in Kenya could have been prevented if the mothers had had check-ups for pregnancy complications. Further, about 9.5 and 18.6 percent of the neonatal deaths during the same period were attributable to lack of ANC visits and inadequate ANC visits, respectively. The figure shows that about 10 percent of neonatal mortalities in Kenya could be attributable to lack of a single TT injection. Unskilled ANC assistance could account for 9 percent of neonatal deaths.

## Discussion

The present study is perhaps the first of its kind to be conducted in Kenya at national level and after the introduction of free maternal and child health care services in mid-2013 in all the first-level public health facilities [16]. Overall, our findings suggest that lack of skilled ANC attendance and no or inadequate ANC visits were the key ANC factors associated with neonatal mortality. ANC visits to a health facility provide the basis for other ANC services.

The results also indicate that a single TT injection to all pregnant women in Kenya could prevent about 10 percent of neonatal deaths in Kenya. Similar studies in Ghana and India and an aggregate-level study in other sub-Saharan countries reported similar findings [1,25,26]. The findings also indicate that availing of check-ups for complications during pregnancy could avert about 38 percent of neonatal deaths in Kenya. Slightly lower estimates were observed in a hospital-based prospective cohort study (2015) conducted in neighboring Uganda that attributed 17.2 percent of neonatal deaths to obstetric complications [49]. One might argue that Uganda has had free maternal health care services in first-level health facilities (following the abolition of user fees) since 2001 [50], long before Kenya followed suit [16], and that could explain the difference in the attributable mortality fractions since more Ugandan women had free access to ANC services.

Our findings have key implications for maternal health care operations in Kenya because they not only validate most of the WHO recommendations [13], but also imply that new strategies such as outreach programmes (such as community or home visits) by skilled nurses to administer ANC services such as check-ups and one TT injection could effectively and significantly reduce neonatal mortalities. This is particularly plausible due to the fact that the NMR among ANC non-attendees was 42.6 per 1000 live births, 4 times higher than among mothers who attended ≥ 4 or more times.

In Figure 2, it appears as if insufficient ANC visits (1–3 ANC visits) accounted for double (18.6 percent) the percentage of neonatal deaths accounted for by zero ANC visits (9.1 percent). This is sufficiently true due to the very few mothers (5 percent) that are in the category of zero ANC visits compared to the many mothers (38 percent) in the 1–3 ANC visits category. It is also most probable that the 5 percent did not seek any ANC because they felt they had no pregnancy complications or symptoms of complications.

Our findings could not explain why two or more TT injections relative to one TT injection did not provide any additional protection to the neonates as shown by the crude and adjusted odds ratios that are not statistically significant. However, a study in India indicated that one and two TT injections equally (equal OR) reduced the risk of neonatal mortality [51]. Given the state of low-resourced settings in SSA, the results reflect that comprehensive population coverage with a single TT injection during the antenatal period could generate a much greater national progress in reducing neonatal deaths in Kenya. However, further research is needed to explore the impact of two TT injections in reducing neonatal deaths in Kenya.

Contrary to our findings, studies have hypothesized the protective effect of consuming ≥ 90 IFA tablets towards newborn survival [1]. Investigating the effectiveness of IFA in a cross-sectional study has key limitations in that iron and folate can easily be obtained in mothers’ daily diets. Further, it is very difficult for the majority of the less educated, rural women to recall the quantity of IFA they bought or the actual number of tablets taken in their last pregnancy. In this study, less than 5 percent took the recommended 90 or more IFA tablets. This study therefore recommends further research on the effectiveness of IFA with a more localized approach linked to maternity hospitals with close follow-up within the counties. This will provide a stronger evidence basis on which appropriate policy adjustments can be implemented.

We note that it is mainly through ANC visits to the health facilities that the ANC services are administered to the women. In addition to the community outreach proposed in this study, we also advocate for further training and appraisal of less skilled health workers such as nursing aids and clinical officers, and refresher training for doctors and nurses. The focus of this training ought to be tailored towards early detection of pregnancy complications and provision of the most effective care to the highest possible numbers, by, for instance, one TT injection instead of two for each expectant mother. Modification of the WHO guidelines can also be done using the best practice approaches to ensure the maximum possible service provision during the fewest visits possible for an expectant mother. For instance, multiple interventions could be combined. Further research on the impact of continued care from antenatal to delivery to the postnatal period by skilled providers could provide a holistic and effective approach towards reducing neonatal deaths and subsequently under-five deaths in the post-Millennium Development Goals (MDG) era.

## Methodological considerations

Due to the introduction of county governments in Kenya, the 2014 DHS data collection and sample size was increased to almost three times more than the previous DHS samples. This provided our study with a comparatively higher statistical power than previous DHS-based studies in Kenya, thus increasing the validity of the results in this study. A key limitation of our study data is that recall bias might not have been completely eliminated. This is especially true for the mothers who gave birth to their last born earlier in the five-year period prior to the survey. Mortalities may have been underestimated due to under-reported deaths.

This study does not confirm the causal association between lack of or inadequate ANC services and neonatal mortality. This is because we could not ascertain the actual cause(s) of deaths among the neonates.

Although this study briefly mentioned the WHO’s recommendations regarding the quality and timing of ANC visits, our data provided only the reported quantity as narrated by the respondents. It is possible that some complex measures of the ANC package such as the BP check or urine or blood analysis results can be misunderstood and misreported by many, especially uneducated mothers. Further, reporting of the IFA tablets intake is likely to be less accurate, so we suggest a follow-up study that can monitor the actual intake or non-intake of the tablets and compare this with the neonatal outcomes.

## Conclusion

Lack of check-ups for complications during pregnancy, insufficient ANC visits, lack of skilled ANC provision and lack of tetanus injection were associated with the risk of neonatal mortality in Kenya. New strategies such as community ANC outreach programmes could supplement the regular ANC visits. Further, training of health workers with a focus on early detection of pregnancy complications and administration of at least one TT injection to all expectant mothers can have quick and significant positive implications towards neonatal survival in resource-limited Kenya.

## Paper context

Inadequate antenatal care is a major reason for the slow decline of neonatal mortality in Kenya. The Division of Reproductive Health (DRH) is currently implementing the Vision 2030 which aims to lower the number of neonatal deaths. However, there is no comprehensive standardized system to evaluate the antenatal care interventions in terms of neonatal survival. We hope our study will provide a basis for continuous countrywide research and contribute to policy developments aimed to improve neonatal survival.
